# BabyByte: Qualitative Research to Inform the Development of an App to Improve Responsive Feeding Practices in Parents of Infants and Toddlers

**DOI:** 10.3390/ijerph20064769

**Published:** 2023-03-08

**Authors:** Amy R. Mobley, Danielle E. Jake-Schoffman, David A. Fedele, Elder Garcia Varela, Jamie Zeldman

**Affiliations:** 1 Department of Health Education and Behavior, University of Florida, Gainesville, FL 32611, USA; 2 Department of Clinical and Health Psychology, University of Florida, Gainesville, FL 32611, USA

**Keywords:** mHealth, technology, pediatric obesity, low-income population, parents, child feeding, infants

## Abstract

Responsive feeding is associated with a reduced risk of childhood obesity. The objective of this qualitative study was to determine parental preferences for mobile health (mHealth) app content and features designed to improve responsive feeding practices. Parents of 0–2-year-old children were interviewed individually. Interview questions were informed by the Technology Acceptance Model, and parents provided feedback on sample app content and features. Interviews were audio-recorded, transcribed verbatim, and coded by two researchers using thematic analysis; responses were compared by parent gender and income. Parents (*n* = 20 fathers, *n* = 20 mothers) were, on average, 33 years old, low-income (50%), identified as non-white (52.5%), and had a bachelor’s degree or higher (62%). Overall, parents were most interested in feeding tips and recipe content, and app features that allowed tracking child growth and setting feeding goals. Fathers were most interested in content about first foods, choking hazards, and nutrition information, while mothers preferred content on breastfeeding, picky eating, and portion sizes. Parents with lower incomes were interested in nutrition guidelines, breastfeeding, and introducing solids. Non-low-income parents preferred information related to food allergies, portion sizes, and picky eating. The findings of this study provide considerations when developing mHealth apps to improve responsive feeding practices in parents.

## 1. Introduction

The first 1000 days (conception to age 2) are critical for obesity prevention [1], yet, there are few effective, sustainable interventions designed for this time period [2]. In fact, one out of four children are already overweight or obese by five years of age [3]. These rates disproportionally affect children from lower-income families [4], continue to increase sharply into adulthood, and are associated with lifelong health-related comorbidities, including cardiovascular disease risk factors [5,6,7,8]. There is a pressing need for interventions that reach large numbers of at-risk children ages 0–2 years because parents [9] and healthcare providers [10] may fail to adequately address early obesity risk. 

Children’s food preferences and eating patterns are established at a young age [11], and these patterns can affect risk factors for obesity and chronic diseases later in life [12]. Unfortunately, many infants and toddlers are fed in ways that predispose them to obesity, such as eating inadequate fruit, vegetables, and iron-rich foods and receiving weaning foods before 4–6 months of age [13]. However, despite the public health need to prevent childhood obesity, there is minimal and varying outreach to parents regarding early optimal feeding practices, and the messages that are conveyed are often inconsistent (e.g., when to introduce first foods) [14]. Further, while mothers are often the main target for childhood obesity prevention interventions, it is recognized that fathers or other family members in the household may influence target behaviors related to early childhood obesity risk [15,16,17,18,19]. Further research is needed to determine the best methods for engaging fathers in family interventions focused on nutrition and health [20]. Therefore, both mothers and fathers are important targets for early childhood obesity prevention outreach. 

In addition, because obesity disproportionally affects children from families with lower incomes, it is important to consider the different preferences and needs of families with lower incomes compared to higher incomes. However, the likelihood of individuals with lower incomes using digital health interventions to improve or support health or dietary behaviors may be lower than individuals with higher incomes [21]. Alternatively, another reason why individuals with lower incomes may be less likely to use digital health technology may be attributed to a real or perceived “digital divide”, although more recent evidence indicates that 76% of lower SES adults report owning a smartphone. However, this proportion is lower compared to middle- (87%) or higher-income adults (97%) [22]. Further inquiry is needed to determine interest in and preferences by parents with lower incomes in using digital technology, such as a child feeding app.

The relationship between parental feeding practices, feeding styles, and children’s dietary intake and weight status has propelled interest in responsive feeding to prevent childhood obesity [23]. Responsive feeding is the process of recognizing a child’s hunger and fullness cues and responding appropriately. In turn, this encourages self-regulation and supports cognitive, emotional, and social development in young children [24]. Implementing responsive feeding from birth helps children maintain their ability to recognize and respond to their hunger and satiety cues [25], thereby allowing better regulation of dietary intake based on physiological needs and decreasing excessive caloric intake and the risk of obesity. Indeed, responsive feeding interventions for parents with infants can curb the development of early childhood obesity [26]. 

When considering intervention strategies, mobile health (mHealth) may be an effective way to promote behavior change related to nutrition and dietary practices using nutrition messaging [27]. To date, however, commercial digital technology tools for children’s healthy eating lack evidence-based content related to dietary and feeding guidance [28], and there is a dearth of tools specifically for feeding young children. Current conflicting messages about child feeding may contribute to confusion among parents and caregivers about what and when to feed their young children and, in turn, lead to inappropriate feeding practices increasing obesity risk [14]. Although there is limited research on mobile phone-delivered information to parents on topics related to child feeding or nutrition, the resources available contain information for breastfeeding or preschool-aged children and do not typically include feeding recommendations regarding the crucial developmental ages in the first 1000 days, including from 0–2 years [29]. Specifically, a systematic assessment of infant feeding apps revealed that although the quality of available apps has improved in the last decade, the credibility and accuracy of content have not improved, with almost half of the available apps containing poor quality, inaccurate, or inadequate information [30]. Innovative approaches to delivering comprehensive and evidence-based feeding messages to parents and caregivers of young children are critically needed. 

The objective of this qualitative study was to determine parents’ preferred content and features for a mHealth app designed to improve responsive feeding practices. Differences between mothers’ and fathers’ preferred content and features (e.g., goal setting) and income status (low-income vs. non-low-income) were also evaluated. 

## 2. Materials and Methods

### 2.1. Participant Eligibility, Recruitment, and Data Collection

The study was approved as exempt by the University of Florida (UF) Institutional Review Board for Human Subjects. Using study flyers and email, parents were recruited through local childcare centers, early Head Start, and community locations serving families with young children. We also partnered with UF HealthStreet, a community engagement program at UF designed to navigate community members to ongoing research projects. We reached out to families who agreed to be contacted for research studies via UF’s Consent2Share program, and we also posted advertisements on social media sites. Parents were eligible if they were at least 18 years of age, able to speak and read in English, had at least one child between 0–2 years old, were responsible for feeding their child at least 50% of the time, and owned or recently owned a smartphone within the last 6 months. Purposeful recruitment was used to include a convenience sample with equal numbers of mothers and fathers and low-income and non-low-income parents. Interviews were conducted until data saturation was reached. Low income was defined as being eligible for certain state or federal nutrition assistance or related programs in the United States (e.g., Supplemental Nutrition Assistance Program) that have income eligibility criteria. After obtaining written informed consent, parents participated in one-on-one semi-structured interviews lasting 45–60 min with a trained researcher and completed a separate demographics and technology use questionnaire. A $25 gift card incentive was provided immediately after the interview.

Interviews were guided by a trained researcher using a semi-structured interview script (File S1, Supplementary Materials) and sample mobile phone screenshots that were shown to the participant on a mobile phone that could be included in a future child feeding app. Questions were based on the Technology Acceptance Model, which draws upon constructs such as perceived usefulness and ease of use, attitudes, and behavioral intentions to predict the actual use of health-related technology [31]. Additional constructs derived from the Theory of Planned Behavior (TPB) and complementary to the TAM were also applied in developing questions within the interview script about the proposed app features and ideas in general to determine perceived usefulness, ease of use, social attitude/subjective norms and behavioral intention for using a child feeding app.

### 2.2. Child Feeding App Development

BabyByte, the name of the proposed mHealth app, was developed using Marvel online prototyping software (marvelapp.com, London, UK, accessed on 5 March 2023) to display potential app features and content on a mobile phone screen during the interview. It was designed to appear as screens from an interactive app on iOS and Android operating systems but was not able to capture direct user input or data. Instead, it served as a visual guide to identify preferred topics and features by parents. The BabyByte sample app screens contained a main menu with proposed feature options: Goal Setting (allows parents to set goals related to child feeding, such as how long a mother may plan to breastfeed or when a parent plans to introduce solid foods); Tracking Baby’s Growth and Milestones (a tool to enter child measures to track growth and also track feeding milestones such as when a child is able to drink from a cup); Parent Discussion Board (an opportunity to connect with other parents with similar feeding questions or concerns); Ask the Expert (connection to a registered dietitian or health professional to answer questions related to child feeding); Surveys & Quizzes (a tool to reinforce content learned and reward parents for submitting and completing questions); Videos (reinforcement of content information and how-to on topics such as introducing or preparing foods, etc.); and More Information (additional electronic resources such as websites and local programs). The features in the app were designed to support behavior change and optimal responsive feeding practices through goal setting, monitoring, social support, reinforcement, and improved self-efficacy.

In addition, a user account example screen and content sections containing feeding topics for three main age groups of children were included as 0–6 months, 6–12 months, and 12–24 months. Age groups were organized based on optimal developmental feeding milestones. Content for the app prototype was based on early childhood obesity prevention information and related features based on priority topics identified by the target population from prior research conducted by the corresponding author [32,33,34] and key strategies from the Robert Wood Johnson Foundation’s (RWJF) Feeding Guidelines for Infants and Young Toddlers: A Responsive Parenting Approach [24]. These topics included when and how to introduce solid foods, how to safely introduce allergenic foods, how to encourage autonomy and self-regulation, and how to increase the variety and healthy food options in a young child’s diet. 

### 2.3. Data Analysis

Interviews were audio-recorded and transcribed verbatim. Transcripts were coded by two research team members using standard word processing software (Microsoft Word) for data extraction and a thematic analysis approach [35]. The research team coded the transcripts independently and met to compare similarities and discrepancies in the coding to reach a consensus. Qualitative crosstab analysis was used to compare responses by gender and income status. Major themes for the overall group of parents and subthemes by parent gender and income were then summarized in descending order of how often mentioned. Verbatim sample quotes from fathers (FA) or mothers (MO) and low-income (L) or non-low-income parents (NL) were labeled accordingly. Descriptive statistics for demographics and responses to technology use questions were also summarized using IBM Corp. Released 2021. IBM SPSS Statistics for Windows, Version 28.0. Armonk, NY, USA: IBM Corp.

## 3. Results

### 3.1. Demographic and Technology Use Characteristics 

Parents (*n* = 20 fathers, *n* = 20 mothers) were, on average, 33 years old, with more than half who were non-white (*n* = 21, 52.5%), non-Hispanic (*n* = 39, 97.5%), had a bachelor’s degree or higher education (*n* = 25, 62%), were employed full-time (*n* = 24, 60%), and were married (*n* = 31, 77.5%) (Table 1). Half of the participants (*n* = 20, 50%) were considered low-income, as indicated by self-reported eligibility for federal or local nutrition assistance or related programs. 

When assessing technology use, most parents reported they own an Apple (*n* = 20, 50%) or Android (*n* = 19, 47.5%) smartphone, had an unlimited data plan (*n* = 25, 67.6%), used apps on their phone more than once per day (*n* = 29, 72.5%), and searched for health information on their phone at least a few times per week (*n* = 26, 65%) (Table 1). When considering the feasibility of using an app to support responsive feeding, the majority (*n* = 34, 85%) of parents indicated that they would be likely or very likely to use it (rating it a 4 or 5 on a scale of 1 to 5). 

### 3.2. Qualitative Results 

Overarching themes resulting from the interviews with parents are summarized in Table 2, with subthemes outlined by parent gender and income comparisons. Most parents, irrespective of gender and income, expressed positive attitudes towards using a child feeding app like BabyByte and indicated it as useful and easy to use and that they would be likely or very likely to use it. Specifically, parents perceived app content options related to feeding tips, recipes, and food allergy guidance and the tracking growth and milestones and setting feeding goals features as most helpful or important to include in a child feeding app, with very few comments regarding features that were not helpful. As one mother shared, “The food tips, and recipes. Because that can help me include some things that I didn’t know, in my… food routine.” (MO-28-L). Most parents also indicated that they were comfortable using all the menu options.

When considering social attitudes or norms, parents indicated that the overall information provided in the app would be beneficial to others. The app’s convenience, reliability, and user-friendliness were also noted as benefits. For example, one mother shared, “To me it’s such wonderful information at your fingertips. It’s just right there, and it’s a good resource to be able to keep track of their eating and their health.” (MO-13-NL) Additionally, one father noted, “Knowing that you have credible, good current information. It’s not just like a Google search. It’s more like a polished professional type of app.” (FA-03-NL) The option to communicate with other parents was also a benefit mentioned by fathers, “I think getting connected to a network of other parents can be a huge benefit for those looking for others to communicate about what are you going through, how’s feeding your child doing, what kind of tips do you have.” (FA-10-L).

When comparing fathers’ and mothers’ preferences for app content, fathers indicated that they were most interested in information about first foods, choking hazards, nutrition information, and food allergies. For instance, one father shared that food allergies were a topic he was most interested in within the app, “Allergies that a baby can have from foods. Because they can get sick then you have reactions and stuff and you don’t want the baby have to go through that. So, the parents get informed about possible things that are big amongst their babies.” (FA-26-L) In comparison, mothers preferred content on breastfeeding, picky eating, and portion sizes. One mother shared that she preferred the content about “…what you can do potentially early on to avoid things down the road of having more picky eating habits.” (MO-18-NL) Another mother noted that she preferred information including “…how much to feed the baby, and all of that stuff. How much on a good day a child should eat, opposed to not overfeeding, and that kind of stuff to me, just matters.” (MO-32-L).

Both fathers and mothers favored the “Tracking Growth or Milestones” and “Setting Goals” features when queried about which feature of the app would be most useful or important to include in a child-feeding app. As one of the fathers shared, “Oh, the tracking my baby. That’s the most customizable thing. All the other stuff, if it’s a hassle, I can go and actually find those things. This is just for my baby.” (FA-07-L) In addition, one of the mothers noted, “One of the things I was most excited about was… the tracking part of it and the milestones… that’s not something we spend a lot of time on.” (MO-19-NL).

When comparing parents’ preferences for app content by income status, parents with lower incomes were interested in information about nutrition guidelines, breastfeeding, and introducing solids. One low-income parent shared that she was interested in content related to “Definitely breastfeeding, I think should be at the highest because why not? Educate more people to know that breast milk, it’s been going on since forever. I think it’s very important.” (MO-32-L) In comparison, parents who were not low-income tended to prefer information related to portion sizes and picky eating. “Probably that all kids are going to be picky and that whole deal, pickiness and how to get your kid to eat something that is new. And then I like the portion video also talking about what they should be eating on a good day.” (FA-40-NL).

When considering the app features, parents with lower incomes perceived the feature “Setting Goals” as the most useful, while non-low-income parents favored “Eating Milestones.” One parent with a lower income noted that “Setting some goals is the most extremely helpful thing out of this whole app, because it allows you to basically set long-term… short-term goals. Things where you are hands on with your baby, where you’re constantly paying attention to everything that they’re doing, everything that in-taking, their growth, all this kind of stuff.” (FA-09-L) While a non-low-income parent shared, “I like the eating milestones and having that in an app, I’m more inclined to do everything.” (MO-23-NL).

Overall, parent participants also indicated that they thought other parents would like the app, with one main caveat of the importance of ensuring that the information is complete and up to date. To encourage further or continued use of the app, parents indicated that the overall layout and design, convenient access, up-to-date content from a reputable and identifiable source, videos, and incentives for completing modules were important features or considerations. As one mother noted, “It definitely would probably be the only useful app. And if University of Florida made it, it’s going to be a really good app.” (MO-22-L) Considering motivators to use the app, another mother added, “If there was a little incentive to use it, that’d be like, yes that’s my new favorite app.” (MO-11-L).

## 4. Discussion

Overall, parents expressed interest in using a child-feeding mobile phone app like BabyByte for accessing feeding-related guidance for children ages 0–2 years. With current parental confusion surrounding early childhood feeding [14,24] and reported nutrition education needs by parents [33,34] for early childhood obesity prevention, an app like BabyByte may be helpful as an intervention option. This is especially true for family members who may be more difficult to reach or less willing to commit to in-person meetings or sessions [36]. 

The app features most frequently mentioned as helpful or important by parents included “Goal Setting” and “Tracking Baby’s Growth or Milestones.” Not surprisingly, Goal Setting has been noted as one of the most commonly applied behavior change techniques of digital interventions, especially those targeting individuals with lower socioeconomic status [37]. In addition, if parents experience confusion related to appropriate child development or milestones, having further support and reassurance could be viewed as beneficial. The use of developmental milestones has been highlighted as an opportunity to reach parents for a range of behaviors [38]. It has been prior noted that almost one out of four parents worry that their child is behind on milestones [39].

While overall preferences for features and content within BabyByte were mostly consistent across parent type and income status, some minor differences in preferences emerged. Overall preferences for app features and content included topics such as breastfeeding, first foods, picky eating, and food allergies. Fathers were often interested in topics that related to safety, such as choking hazards and food allergies. This may reflect an instinctive role of a father to serve as a provider or “protector” of his child [40]. In comparison, mothers more frequently mentioned the importance of topics such as breastfeeding and picky eating, likely because it is a behavior in which they may be more engaged. However, emerging technologies and interventions target fathers on topics such as breastfeeding to encourage support for their partners [41].

There were some minor variations in content preferences between low-income and non-low-income parents, which is consistent with prior research assessing the nutrition education needs of parents in relation to child feeding [34]. In addition, there were minimal differences when comparing low-income and non-low-income parents’ preferences for the app features. While the lack of technology-based interventions and evaluation of acceptability for lower socioeconomic families has been acknowledged prior [42], these sentiments were not shared by parents with lower incomes in this study. 

When considering the use of technology by mothers and fathers related to infant feeding, researchers determined that more mothers used the internet when seeking child-feeding information compared to fathers [20]. In addition, while fewer fathers were using the internet to seek child health or feeding-related information compared to mothers [20], it is possible that online information is not currently targeted or tailored towards fathers and, therefore, less likely for fathers to seek or find this information [43]. It has been recommended to increase engagement with fathers in early obesity prevention, especially during the first 1000 days [15]. Some fathers in the study indicated that the app would be beneficial by providing the opportunity to connect or communicate with other parents. Other child development focus group reports have recommended this aspect of social support, especially for first-time parents and fathers who are often not the primary target for parent support groups [38]. For example, a prior pilot initiative established the feasibility of engaging fathers in a perinatal intervention providing an example of how digital technology can serve as a viable mechanism to target fathers [44]. The current study provides further evidence of interest, positive attitudes, and behavioral intentions from fathers of young children in using an app to improve responsive child-feeding practices.

Overall, while prior studies have explored the use of technology for family interventions as it relates to child health, mHealth tools are less frequently used, if at all, and results have been mixed [36]. Thus, it is important to also evaluate and consider the different components of mHealth tools and how they support behavior change either individually or synergistically, such as through the use of the Multiphase Optimization Strategy (MOST) [45]. At a minimum, as it relates to child development, it has been suggested to provide clear and concise recommendations to parents without overwhelming them, offering “how-to” guidance, providing support as recommendations are implemented, and giving options and alternatives for diverse needs [38].

### Limitations

While the study has many strengths, such as the inclusion of both mothers and fathers and low-income parents, as well as providing qualitative considerations for using a child-feeding-related mobile phone app, there are some limitations. A convenience sample of parents recruited for the study was from one geographic region or state (Florida) of the USA and tended to have higher education levels than the general population; therefore, their perceptions may not be widely transferable, and the findings may not be generalizable. Data were self-reported, and questions were based on an initial app prototype that was not yet fully developed or functional. It is unknown how and if parents would actually use the app in their daily routine.

## 5. Conclusions

The findings of this study are important when considering the development of future mHealth tools for parents of children ages 0–2 years to improve responsive feeding practices and prevent early childhood obesity. While parents of children ages 0–2 years were in favor of using an app to support child feeding, some nuanced differences emerged in terms of preferred content or features. Thus, having various features, topics, and personalization available could allow choices and tailoring when developing mHealth tools to promote and support optimal child-feeding behaviors for parents. Future research is needed to determine the usability and feasibility of a child feeding app by parents of children ages 0–2 years and to evaluate the impact on parental feeding behaviors and associated child health and nutrition outcomes.

## Figures and Tables

**Table 1:** Demographic Characteristics and Technology Use of Parents with Children Ages 0–2 Years Participating in an Interview about a Mobile Phone Child Feeding App.

Demographic Characteristics	Overall (*n* = 40)	Mothers (*n* = 20)	Fathers (*n* = 20)	Low-Income (*n* = 20)	Non-Low-Income (*n* = 20)
Age, y, mean (SD)	33.0 (6.0)	33.3 (6.6)	31.5 (8.7)	31.2 (6.6)	34.8 (4.9)
Race, % (*n*)					
White	47.5 (19)	50.0 (10)	45.0 (9)	30.0 (6)	65.0 (13)
Black or African American	30.0 (12)	30.0 (6)	30.0 (6)	50.0 (10)	10.0 (2)
American Indian/ Alaskan Native	2.5 (1)	0.0 (0)	5.0 (1)	0.0 (0)	5.0 (1)
Asian	12.5 (5)	10.0 (2)	15.0 (3)	10.0 (2)	15.0 (3)
More than one race	5 (2)	5.0 (1)	5.0 (1)	5.0 (1)	5.0 (1)
Other	2.5 (1)	5.0 (1)	0.0 (0)	5.0 (1)	0.0 (0)
Ethnicity, non-Hispanic, % (*n*)	97.5 (39)	100.0 (20)	95.0 (19)	95.0 (19)	100 (20)
Income Level, Low-income % (*n*)	50.0 (20)	50.0 (10)	50.0 (10)	100 (20)	0.0(20)
Highest level of education, % (*n*)					
Less than high school	2.5 (1)	5.0 (1)	0.0 (0)	5.0 (1)	0.0 (0)
High school graduate	15.0 (6)	5.0 (1)	25.0 (5)	30.0 (6)	0.0 (0)
Some college or technical school	7.5 (3)	10.0 (2)	5.0 (1)	10.0 (2)	5.0 (1)
Associate degree	12.5 (5)	10.0 (2)	15.0 (3)	15.0 (3)	10.0 (2)
Baccalaureate degree or more	62.5 (25)	70.0 (14)	55.0 (11)	40.0 (8)	85.0 (17)
Employment status, % (*n*)					
Stay-at-home-parent	15.0 (6)	25.0 (5)	5.0 (1)	25.0 (5)	5.0 (1)
Employed part-time	12.5 (5)	20.0 (4)	5.0 (1)	25.0 (5)	0.0 (0)
Employed full time	60.0 (24)	50.0 (10)	70.0 (14)	30.0 (6)	90.0 (18)
Not Employed	12.5 (5)	5.0 (1)	20.0 (4)	20.0 (4)	5.0 (1)
Marital status, % (*n*)					
Single, never married	15.0 (6)	15.0 (3)	15.0 (3)	30.0 (6)	0.0 (0)
Married	77.5 (31)	80.0 (16)	75.0 (15)	60.0 (12)	95.0 (19)
Divorced	2.5 (1)	0.0 (0)	5.0 (1)	5.0 (1)	0.0 (0)
Living with Partner	5.0 (2)	5.0 (1)	5.0 (1)	5.0 (1)	5.0 (1)
**Other Demographics**					
Age of child, % (*n*)					
0–6 months	17.5 (7)	20.0 (4)	15.0 (3)	25.0 (5)	10.0 (2)
7–12 months	22.5 (9)	20.0 (4)	25.0 (5)	25.0 (5)	20.0 (4)
13–24+ months	60.0 (24)	60.0 (12)	60.0 (12)	50.0 (10)	70.0 (14)
Number of children, % (*n*)					
1 child	40.0 (16)	25.0 (5)	55.0 (11)	35.0 (7)	45.0 (9)
2–3 children	55.0 (22)	65.0 (13)	45.0 (9)	65.0 (13)	45.0 (9)
4+ children	5.0 (2)	10.0 (2)	0.0 (0)	0.0 (0)	10.0 (2)
**Technology Use Questions**					
Smartphone Operating System, % (*n*)					
Android	47.5 (19)	55.0 (11)	40.0 (8)	55.0 (11)	40.0 (8)
Apple	50.0 (20)	40.0(8)	60.0 (12)	40.0 (8)	60.0 (12)
Other	2.5 (1)	5.0 (1)	0.0 (0)	5.0 (1)	0.0 (0)
Data Plan, unlimited-yes, % (*n*)	62.5 (25)	88.9 (16)	47.4(9)	60.0 (12)	65.0 (13)
Frequency of using a smartphone to search for health-related information					
Never	5.0 (2)	0.0 (0)	10.0 (2)	5.0 (1)	5.0 (1)
Rarely	7.5 (3)	0.0 (0)	15.0 (3)	5.0 (1)	10.0 (2)
Couple of times a month	22.5 (9)	20.0 (4)	25.0 (5)	20.0 (4)	25.0 (5)
Couple of times a week	37.5 (15)	35.0 (7)	40.0 (8)	30.0 (6)	45.0 (9)
Once a day	5.0 (2)	5.0 (1)	5.0 (1)	10.0 (2)	0.0 (0)
More than once per day	22.5 (9)	40.0 (8)	5.0 (1)	30.0 (6)	15.0 (3)
Frequency of using Apps, % (*n*)					
Never	2.5 (1)	0.0 (0)	5.0 (1)	0.0 (0)	5.0 (1)
Couple of times a month	5.0 (2)	10.0 (2)	0.0 (0)	10.0(2)	0.0 (0)
Couple of times a week	10.0 (4)	10.0 (2)	10.0(2)	15.0 (3)	5.0 (1)
Once a day	10.0 (4)	20.0 (4)	0.0 (0)	10.0 (2)	10.0 (2)
More than once per day	72.5 (29)	60.0 (12)	85.0 (17)	65.0 (13)	80.0 (16)
Likelihood of using a Child Feeding App, Mean (SD) *	4.6 (0.7)	4.6 (0.7)	4.6 (0.8)	4.8 (0.5)	4.4 (0.9)

* Scored on a scale from 1 to 5, with 1 as not likely and 5 as very likely.

**Table 2:** Qualitative interview question topics and response themes and subthemes from parents (*n* = 40) of children ages 0–2 years old to guide the development of a mobile phone application (mHealth app) containing early child feeding and obesity prevention educational content.

Interview Question Topic	Theoretical Construct	Response Themes *	Response Subthemes * by Parent Type		Response Subthemes * by Income Status	
			Fathers	Mothers	Low-Income	Non-Low-Income
Content or app features most important or helpful to include	Perceived Usefulness (TAM)	All content and features important Fun food tips & recipes	Fun food tips and recipes: first foods, choking hazards, nutrition guidelines Tracking baby’s milestones/growth Food allergies	Fun food tips & recipes: breastfeeding, picky eating, portion sizes Tracking baby’s milestones/growth	Fun food tips & recipes: nutrition guidelines, breastfeeding, first foods Setting goals	Fun food tips & recipes: food allergies, portion sizes, picky eating Tracking baby’s eating milestones
Benefits of using an app for child feeding	(Social) Attitude & Subjective Norm (TPB/TAM)	Overall information provided	Overall information Communicate with other parents	Overall information Convenient Reliable	Overall information Fun food tips & recipes Convenient Reliable	Overall information Helpful information User friendly
Features that would increase the use of a child-feeding app	Behavioral Intention (TPB/TAM)	All features important Overall layout/design	Ask the expert Fun food tips & recipes	Videos Incentives	Fun food tips & recipes Source	Overall design/layout Source Videos

* In descending order of frequency mentioned by parents. TAM—Technology Acceptance Model. TPB—Theory of Planned Behavior.

## Data Availability

Qualitative data are available upon reasonable request from the authors.

## Supplementary Materials

The following supporting information can be downloaded at: https://www.mdpi.com/article/10.3390/ijerph20064769/s1, File S1: Semi-Structured Interview Script (amended to include questions analyzed for manuscript).
