# Supplementary material for: BabyByte: Qualitative Research to Inform the Development of an App to Improve Responsive Feeding Practices in Parents of Infants and Toddlers
Source: Int J Environ Res Public Health. 2023 Mar 8;20(6):4769. doi: 10.3390/ijerph20064769 (PMC10049234; doi:10.3390/ijerph20064769)
Supplement: Supplementary file 1 [file ijerph-20-04769-s001.zip › ijerph-2223072-supplementary.pdf]

File S1. Semi-Structured Interview Script (amended to include questions analyzed for manuscript)

Introduction

** Start recorder ** [Do not transcribe introduction]

Hello, My name is ____________.

Thank you for taking the time out of your day to speak with me.

We are looking to gather feedback from parents who have at least one infant or toddler, about their opinions about using technology on their cell phone like certain health related apps and videos that contain food and health information to guide us in the development of our child feeding app. Please share any/all of your opinions, as there are no right or wrong responses, and all of your responses will remain confidential and will not be linked to your personal information. I am just here to ask questions, I want to listen, and make sure you have a chance to share. Also, do not feel pressured to answer every question. But know that I am genuinely interested in what you have to say.

To make sure I don't miss anything that is said I will be taking notes and recording the information. If you have more than one child, we are asking these questions as they relate to your infant or toddler.

Ice Breaker
What is your favorite thing to do with your child(ren)?

Key Questions based on prototype and examples

We are going to show you examples of ideas of items that could be included in an app to help parents who have questions about feeding their baby or toddler. When thinking about your answers, please think about how helpful the ideas or options could be to you or other parents with a baby or toddler.

Features and Sample Content - *show examples using Marvel [interviewer: for each item as applicable, ask participant about how important or helpful the feature or content is to include and which one would be most helpful vs. least helpful]*

a. Profile set up
    a. Personalized child development/feeding information based on age of child (upon entering) or, general select topics that are age range or topic focused
    b. Options to upload photos of baby's progress
b. Baby and toddler friendly meals/recipes, feeding tips – organized by age group (0-6 months, 6-12 months, 12-24 months)
c. Goal setting related to feeding (breastfeeding, introducing new foods, mealtime challenges)
d. Monitoring/tracking - first food, child's growth, feeding/breastfeeding
e. Parent discussion board - group/community sharing
f. Ask an expert – connect with a Registered Dietitian or health professional
g. Quizzes and Surveys
    a. Rewards: real vs. "badge"
h. More information: links to more information or referrals

Questions based on Technology Acceptance Model (TAM) and Theory of Planned Behavior (TPB) about proposed app

Perceived Usefulness (TAM)

1. Of the ideas or options shown, which do you think was the most helpful or important to include? Why?
2. Which do you think was the least helpful? Why?

Perceived Ease of Use (TAM)

3. Of the examples shown, which would you be most comfortable with using? Why?

(Social) Attitude & Subjective Norm (TPB/TAM)

4. What would be the benefits for using an app for child feeding information? What might be some drawbacks or things to consider?
5. How do you think other parents would feel about using the app?

Behavioral Intention (TPB/TAM)

6. How likely would you be to use a child feeding app?
   (*on scale from 1 to 5 as 1 as not likely and 5 as very likely)*
7. What features might further increase your use of the app?

Ending Question

8. Is there anything else that you would like to suggest or share?

Thank you for taking the time to join me today. The information you have shared today will help develop future programs and technologies for parents with babies and toddlers.

** Stop Recorder **
